# Platinum Single Atoms Strongly Promote Superoxide Formation in Titania‐Based Photocatalysis – Platinum Nanoparticles Don't

**DOI:** 10.1002/smll.202412097

**Published:** 2025-02-16

**Authors:** Yue Wang, Siming Wu, Giorgio Zoppellaro, Zdeněk Baďura, Patrik Schmuki

**Affiliations:** ^1^ Department of Materials Science and Engineering Chair for Surface Science and Corrosion (WW4‐LKO) Friedrich‐Alexander‐Universität Erlangen‐Nürnberg Martensstraße 7 91058 Erlangen Germany; ^2^ Czech Advanced Technology and Research Institute CATRIN Regional Centre of Advanced Technologies and Materials (RCPTM) Palacky University in Olomouc Šlechtitelů 11 Olomouc 78371 Czech Republic; ^3^ CEET Nanotechnology Centre VŠB–Technical University of Ostrava 17. listopadu 2172/15 Ostrava‐Poruba 70800 Czech Republic

**Keywords:** degradation, photocatalysis, Pt single atoms, superoxide, titanium dioxide

## Abstract

The selective reduction of molecular oxygen to superoxide is one of the key reactions in electrochemistry and photocatalysis. Here the effect of Pt co‐catalysts, dispersed on titania, either as single atoms or as nanoparticles, on the photocatalytic superoxide (^•^O_2_^−^) formation in O_2_ containing solutions is investigated. The ^•^O_2_^−^ formation is traced by nitroblue tetrazolium (NBT) assays and in detail by EPR measurements using TEMPO as ^•^O_2_^−^ radical scavenger. The results show that the photocatalytic formation rate of ^•^O_2_^−^ on titania can strongly be enhanced by using Pt single atoms as a co‐catalyst, whereas Pt nanoparticles hardly exhibit any accelerating effect. This finding is of considerable significance regarding photocatalytic degradation and photocatalytic oxidative synthesis processes.

## Graphical abstract

Platinum single atoms (Pt SAs) strongly catalyze the photocatalytic oxygen reduction reaction to superoxide (•O₂⁻) on rutile TiO₂, whereas platinum nanoparticles (Pt NPs) exhibit little to no accelerating effect. This highlights a remarkable example of speciation‐specific selectivity in single‐atom catalysis.

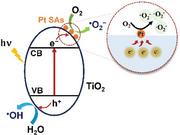

## 1 Introduction

Titanium dioxide (TiO_2_) based photocatalyst has over the past 40 years attracted wide interest, due to its stability against photocorrosion, non‐toxicity, and low cost.^[^ ^1^ ^]^ Photocatalytic reactions on modified and unmodified TiO_2_ are widely used in pollutant degradation^[^ ^2^ ^]^ and other oxidation processes of organic material^[^ ^3^ ^]^ that target the upcycling of low‐value to high‐value products.

In these photocatalytic processes, super band gap light is absorbed by TiO_2_, and the resulting electron‐hole pairs are separated and migrate on the conduction and valence bands to the semiconductor surface, where they can react with red‐ox species in the environment. In aqueous environments, photoelectrons can reduce H^+^ to H_2_ and holes may oxidize water (or other hole capture agents, namely so‐called sacrificial agents).^[^ ^4^ ^]^ In an oxygen‐containing aqueous solution, photogenerated electrons preferentially react with oxygen in the solution, more specifically with O_2_ molecules adsorbed on the catalyst surface, typically to either superoxide radicals (1) or peroxide species (2).^[^ ^5^ ^]^ The single‐electron transfer reaction to superoxide radicals can be represented as a one‐electron transfer:

(1) $$O_{2}+e^{-}\to •{O_{2}}^{-}$$

whereas a two‐electron process yields peroxide according to Equation (2):

(2) $$O_{2}+2e^{-}+2H^{+}\to H_{2}O_{2}$$

Equation (2) can take place directly or as a 2‐step process where superoxide (formed in equation (1)) can dismutate either spontaneously or catalytically to hydrogen peroxide (H_2_O_2_):

(3) $$2^{•}{O_{2}}^{-}+2H^{+}\to H_{2}O_{2}+O_{2}$$

The generation of these reactive oxygen species (ROS), in particular the superoxide radical (^•^O_2_^−^), plays a pivotal role in the photocatalytic activity of TiO_2_.^[^ ^6^, ^7^ ^]^ In recent years, an increasing number of studies have reported the use of photocatalytic processes for the formation of H_2_O_2_, also in view of the industrial replacement of the anthraquinone process.^[^ ^8^ ^]^ The preferential formation of superoxide is much less investigated, although ^•^O_2_^−^ is often regarded as the key in many oxidative pathways in titania photocatalysis.^[^ ^9^ ^]^ Moreover, superoxide is not only beneficial for the destruction of unwanted pollutants but represents a valuable oxidant in the synthesis of various organic compounds.^[^ ^10^ ^]^

In photocatalytic uses of titania surfaces, often the electron transfer to a reactant and thus the rate‐determining step can be accelerated by so‐called co‐catalysts. These are frequently noble metal nanoparticles, such as Pt and Pd that can drastically accelerate the kinetics of the charge transfer reaction. This is particularly the case for photocatalytic H_2_ generation.^[^ ^11^ ^]^ For the oxygen reduction reaction, bulk Pt is usually promoting a four‐electron reaction (O_2_ + 4e^−^ + 4H^+^ → 2H_2_O).^[^ ^12^ ^]^ Only under specific reaction conditions (namely mildly alkaline pH and selected applied potential) O_2_ reduction toward superoxide has been reported.^[^ ^6^, ^13^ ^]^

In photocatalysis, recently the use of Pt single atoms (SAs) catalysts – to replace Pt nanoparticles (NPs) as co‐catalysts – has attracted wide interest in view of drastically accelerated HER. However, the use of Pt SAs in photocatalysis not only provides the ultimate utilization of precious metal but also is able to open up “unusual” reaction pathways.^[^ ^14^ ^]^ For example, Pt SAs have been shown to effectively promote photoelectrochemical (PEC) water oxidation, a reaction in which bulk Pt (or nanoparticles) are ineffective.^[^ ^15^ ^]^

Therefore, in the present work we explore, if there is any benefit of the use of Pt SAs as co‐catalysts on the photocatalytic formation of superoxide. We study Pt SAs decorated rutile surfaces for the formation of superoxide by a classic optical assay (based on the reaction of nitroblue tetrazolium (NBT) with ^•^O_2_^−^) and in more detail using continuous wave (CW) EPR technique under in‐situ light irradiation (using TEMPO as a radical scavenger). We find that Pt SAs on titania rutile powder in an oxygenated aqueous environment strongly accelerate the kinetics of the photocatalytic superoxide (^•^O_2_^−^) formation. Remarkably, Pt nanoparticles used as a co‐catalyst do not (or only minorly) have a catalytic effect on the superoxide production.

## 2 Results and discussion

**Figure 1:**
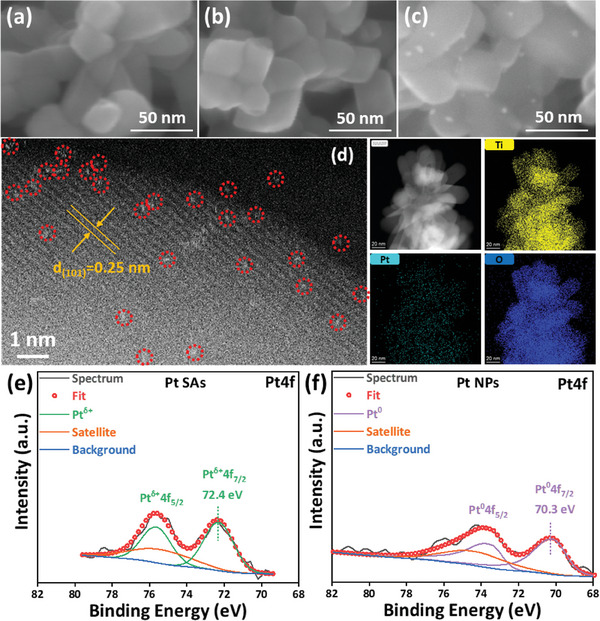
SEM images of a) bare rutile powders, b) rutile powders decorated with Pt SAs by reactive deposition from 0.02 mm H_2_PtCl_6_, and c) rutile powders decorated with Pt NPs by photodeposition from 0.05 mm H_2_PtCl_6_. d) HAADF‐STEM image with energy‐dispersive X‐ray (EDX) mapping for different elements on the rutile powders after reactive deposition from 0.02 mm H_2_PtCl_6_. XPS Pt4f spectra (fitting included) of rutile powders: e) after reactive deposition from 0.02 mm H_2_PtCl_6_ and f) after photodeposition from 0.05 mm H_2_PtCl_6_.

In the first step we decorate commercial rutile nanoparticles either with Pt NPs using classic photodeposition^[^ ^16^, ^17^ ^]^ or with Pt SAs using a reactive SAs deposition method described in the experimental section and in detail in refs.[18] **Figure** **1** shows SEM images of the TiO_2_ rutile powders used in this work before (Figure 1a) and after loading with either Pt SAs (Figure 1b) as well as loading with Pt NPs (Figure 1c). From Figure 1a it is apparent that the rutile nanoparticles have an average size of ≈30 nm and XRD (Figure S1d, Supporting Information) confirms their rutile phase (see JCPDS Card No: 21–1276). For reactive Pt deposition of SAs onto the rutile crystals (Figure 1b) there is no noticeable change in TiO_2_ surface morphology (see also Figure S1a,b, Supporting Information), and, as expected for SAs, no sign of Pt nanoclusters or nanoparticles are observed on the TiO_2_ surface. For photodeposition (Figure 1c and Figure S1c, Supporting Information), as expected, SEM images show clearly visible Pt nanoparticles of a few nm in diameter that are regularly decorated on the rutile crystallites. Note that also XRD patterns for Pt loaded samples (Pt SAs or Pt NPs) show no detectable Pt crystallites, i.e., only the diffraction patterns of rutile TiO_2_ can be identified (Figure S1d, Supporting Information).

For the sample loaded with reactive deposition, the presence of Pt as SAs on the TiO_2_ surface can clearly be revealed through High‐Angle Annular Dark‐Field Scanning Transmission Electron Microscopy (HAADF‐STEM) images and XPS. Figure 1d shows a HAADF‐STEM image after the rutile crystals were decorated from a 0.02 mm H_2_PtCl_6_ solution. The image shows well‐dispersed Pt atoms (examples are circled in red) decorated on a rutile lattice (d = 0.25 nm, consistent with the *d*‐spacing in rutile (101)^[^ ^19^ ^]^) with a Pt SAs density of ≈3.9 ×10^5^ µm^−2^. The Pt single atom density was obtained by evaluating a series of HAADF‐STEM images – this result is well in line with previous studies.^[^ ^20^ ^]^

A first assessment of the Pt loading and the chemical state of the deposited Pt were then obtained from XPS. In the case of SAs decorated sample in Figure 1b, the Pt4f spectra exhibit a doublet with the Pt4f _7/2_ and Pt4f_5/2_ at ≈ 72.4 and 75.7 eV (Figure 1e). This observation is consistent with Pt species coordinated with oxygen atoms of the TiO_2_ surface, where the peak position corresponds to a formal charge (δ^+^) on Pt^δ+^ with a value of δ ≈ 2.^[^ ^21^ ^]^ In contrast, the NPs decorated sample in Figure 1c shows a doublet located at ≈70.3 and 73.7 eV (Figure 1f), which is typical of Pt^0^ in a metallic nanoparticle configuration.^[^ ^16^, ^22^ ^]^ Spectra for some variation in the reactive deposition and photodeposition conditions are shown in Figure S2a (Supporting Information), with details of the fitting procedure in Figure S2b–d (Supporting Information).

Table S1 (Supporting Information) gives an evaluation of the Pt loading on the rutile powders from XPS and atomic absorption spectroscopy (AAS) analyses. Evidently, the Pt loading from the 0.02 mm precursor SAs sample (Figure 1b) and the Pt deposited as NPs (Figure 1c) yield approximately the same overall Pt loading. Additional samples with higher and lower SAs loading and higher Pt NPs loading are also included.

**Figure 2:**
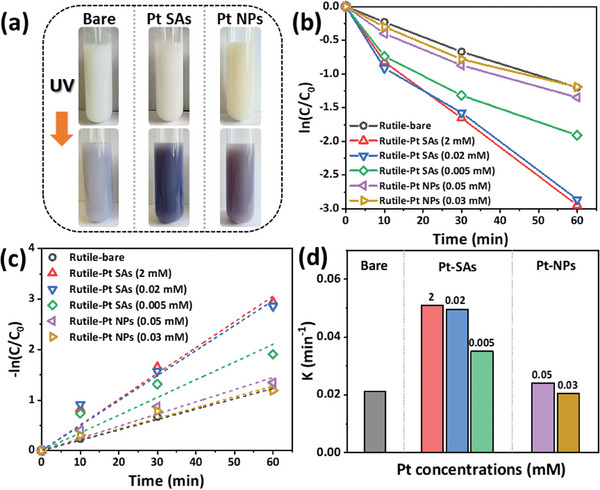
a) Photographs of various suspensions and after NBT assay executed for 10 min. b) Photodegradation tests carried out in air with various samples. The degradation rate constants are carried out by c) linear fitting and shown in d).

We then used different Pt‐loaded and non‐loaded rutile samples for photocatalytic experiments. In order to test for ^•^O_2_^−^ formation, we performed nitroblue tetrazolium (NBT) assays which is a comparably straightforward color indicator reaction for the presence of ^•^O_2_^−^ species^[^ ^23^ ^]^ (see experimental section for details). The test leads, in the presence of ^•^O_2_^−^, to NBT decay and the formation of blue NBT‐Formazan (**Figure** **2**a). Evidently, illumination of the TiO_2_ results for all surfaces in a positive test, but clearly, the activity for the generation of ^•^O_2_^−^ (the depth of blue coloration) follows the order of SAs>>NPs>bare TiO_2_. Spectroscopically one can follow the decay of NBT by its UV‐peak at 260 nm. Figure S3 (Supporting Information) shows UV–vis spectra taken after different UV illumination times (10, 30, 60 min) of the suspended photocatalyst in a 4% 2‐propanol solution containing 40 µm NBT – here 2‐propanol serves as a hole capture agent. Figure 2b presents decay curves for all samples (evaluated at 260 nm from the UV spectra in Figure S3, Supporting Information). The data clearly demonstrate that the presence of Pt SAs co‐catalyst strongly increases the NBT decay rate, with efficiency improving at low concentrations (0.005 mm) and saturating between 0.02 and 2 mm. In contrast, Pt NPs decorated samples exhibit a decay rate similar to that of bare rutile. A linear fitting of the data (assuming a first‐order decay mechanism) is shown in Figure 2c with the corresponding rate constants in Figure 2d. From these experiments, remarkably, the decoration of rutile with Pt NPs has only a very minor accelerating effect on the photocatalytic ^•^O_2_^−^ production, whereas for the Pt SAs a strongly improved ^•^O_2_^−^ production rate can be found.

To elucidate the process of O_2_ reduction in more detail, we carried out EPR measurements using 2,2,6,6, tetramethylpiperidine‐*N*‐oxyl radical (TEMPO) as reactive spin species. Such nitroxide stable radicals are well established as radical scavengers^[^ ^24^ ^]^ and have been extensively used as probes for reactive oxygen species.^[^ ^25^ ^]^ Nitroxides can catalyze superoxide radical anion (^•^O_2_^−^) dismutation by two different pathways − a reductive process and/or oxidative reactions.^[^ ^26^ ^]^ Literature reports show that nitroxides shuttle among three oxidation states and piperidine derivatives, such as 2,2,6,6, tetramethylpiperidine‐*N*‐oxyl radical (TEMPO), are readily oxidized by protonated superoxide, ^•^OOH, a reactive molecule that easily forms from interaction of H^+^(water) with ^•^O_2_^−^, and by ^•^OH radical, to yield diamagnetic (EPR silent) oxoammonium cations.^[^ ^27^ ^]^ We therefore used the EPR technique (X‐band) in combination with TEMPO radical to probe the proclivity of the various Pt‐loaded rutile catalysts for the generation, during light irradiation, of ROS species from O_2_ in water (e.g., ^•^O_2_^−^, ^•^OOH and ^•^OH).^[^ ^28^ ^]^

**Figure 3:**
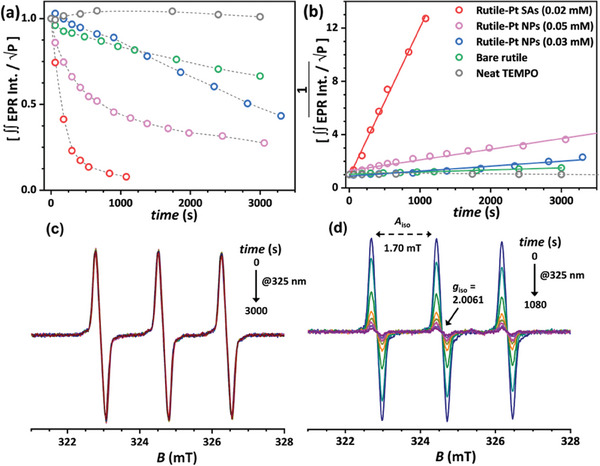
a) The decrease of the TEMPO radical EPR signal, recorded in oxygen containing water solution at *T* = 293 K, versus irradiation time (@325 nm). The *y*‐axis (∫∫ EPR Intensity/√P) indicates the variation of the double integrated signal intensities divided by square root of applied power (√P). The values have been normalized against the value obtained in dark conditions (*t* = 0). Gray‐circles, neat TEMPO in water; Green‐circles, bare rutile and TEMPO in water; Blue‐circles, rutile‐Pt NPs (0.03 mm) and TEMPO in water; Magenta‐circles, rutile‐Pt NPs (0.05 mm) and TEMPO in water; Red‐circles, rutile‐Pt SAs (0.02 mm) and TEMPO in water. b) Analysis of the TEMPO EPR signal loss by second‐order kinetic using the dataset shown in panel (a). Panels c,d) show the evolution versus time of the X‐band EPR spectra of neat TEMPO radical (c) and TEMPO with rutile‐Pt SAs (0.02 mm) catalyst (d) recorded in dark conditions (*t* = 0 s) and during in situ UV‐light irradiation @325 nm (*t* > 0). Experimental parameters: (c) 9.0824 GHz frequency, 0.90 mW applied power and (d) 9.0802 GHz frequency and 0.30 mW applied power. Then, 0.03 s time constant, 0.3 mT modulation width, 1 min acquisition time and *T* = 293 K.

The concentration of TEMPO radical was kept constant in all experiments (1.95 × 10^−4^ m), with and without the addition of TiO_2_ catalysts (bare rutile and variously Pt loaded rutile samples). Under our experimental conditions, and under continuous in situ UV irradiation (@325 nm, 40 mW cm^−2^), when the neat TEMPO radical was dissolved in oxygen‐containing water and in the absence of TiO_2_ catalysts, no significant loss of the radical EPR signal at 293 K was observed, even after prolonged irradiation time, as shown in the plot of **Figure** **3**a (gray circles) and Figure 3c. Therefore, the concentration in the solution of TEMPO, in the form of an active radical scavenger, remained nearly constant throughout the light‐irradiation time probed (50 min). Figure 3c shows the spectra overlay of the TEMPO EPR signals recorded in water, from dark conditions (*t* = 0 min) to 50 min of continuous light irradiation (@325 nm). The presence of Pt‐loaded rutile catalysts in the water suspensions led to a decay of the TEMPO radical under light irradiation, indicating that reactive oxygen species were effectively formed. Figure 3a shows the behavior observed for bare rutile (green circles) where only 44% of TEMPO radical was lost (oxidized) after 50 min @325 nm. In the presence of the NP‐decorated TiO_2_, a slightly faster decay was observed; for Pt NPs (0.03 mm) 50% of TEMPO radical was oxidized in 47 min @325 nm (Figure 3a, blue circles), and for Pt NPs (0.05 mm), 72.5% TEMPO radical was oxidized in 51 min @325 nm (Figure 3a, magenta circles). Most notably, for the Pt SA loaded TiO_2_ sample, Pt SAs (0.02 mm), the far highest rate to produce reactive oxygen species was observed with 99.2% of TEMPO radical oxidized in just 18 min of irradiation (Figure 3a, red circles).

Figure 3d shows the fast disappearance of the TEMPO radical EPR signal recorded under irradiation, in the presence of the Pt SAs‐loaded rutile sample. Although the TEMPO radical is known to be highly sensitive to the presence of the superoxide radical, basically several ROS species (^•^O_2_^−^, ^•^O_2_H, ^•^OH) may contribute to the observed decrease of the EPR signal associated with the TEMPO signal.^[^ ^29^ ^]^ In this context, however, it is important to note that the NBT assay is quite robust in its specificity toward superoxide –, i.e., NBT decay takes place in the presence of superoxide but not in the presence of peroxides. Figure S4 (Supporting Information) shows according to reference experiments with NBT in the presence of H_2_O_2_ where no alterations of NBT can be observed under any of the investigated conditions. Thus, the effects observed in EPR can be reliably attributed to the selective formation of superoxide.

Quantitative analysis of the TEMPO radical disappearance for the various TiO_2_ catalysts is given in Figure 3b; the best fitting results of the experimental data suggest that the TEMPO radical reacts via a 2nd order kinetic process. Bare rutile nanopowder gives a value for the second order rate constant (*k*) for the TEMPO signal disappearance of 0.81 M^−1^ s^−1^ (*k* = k_EPR_ /1.95 × 10^−4^ M = 1.58 × 10^−4^ s^−1^/ 1.95 × 10^−4^ M, *R* ^2^ = 0.99, green circles and green fitting line), rutile‐Pt NPs (0.03 mm) exhibits *k* = 1.91 M^−1^ s^−1^ (*k* = k_EPR_ /1.95 × 10^−4^ M = 3.73 × 10^−4^ s^−1^/ 1.95 × 10^−4^ M, *R* ^2^ = 0.94, blue circles and blue fitting line), rutile‐Pt NPs (0.05 mm) shows *k* = 4.11 M^−1^ s^−1^ (*k* = k_EPR_ /1.95 × 10^−4^ M = 8.02 × 10^−4^ s^−1^/ 1.95 × 10^−4^ M, *R* ^2^ = 0.97, magenta circles and magenta fitting line), being the rutile‐Pt SAs (0.02 mm) the most active material, with *k* = 61.54 M^−1^ s^−1^ (*k* = k_EPR_ /1.95 × 10^−4^ M = 1.20 × 10^−2^ s^−1^/ 1.95 × 10^−4^ M, *R* ^2^ = 0.99, red circles and red fitting line). In these TEMPO decay processes second‐order kinetics), the half‐life (*t*_1/2_ = 1/(*k*[TEMPO]_0_)), corresponds to ≈6331 s for neat TEMPO under constant UV irradiation, ≈2685 s for rutile‐Pt NPs (0.03 mm), ≈1248 s for rutile‐Pt NPs (0.05 mm), and ≈83 s for rutile‐Pt SAs (0.02 mm).

**Figure 4:**
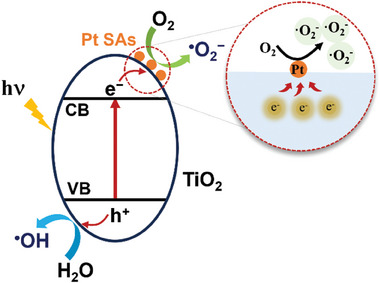
Schematic illustration of the photocatalytic superoxide generation mechanism.

The photocatalytic reaction mechanism is schematically illustrated in **Figure** **4**. Under light illumination, photogenerated electrons are captured by Pt SAs, where they react with O_2_ to form ^•^O_2_^−^ radicals, driving the formation of reactive oxygen species. Simultaneously, photogenerated holes react with H_2_O to produce ^•^OH radicals, contributing to the charge separation process. In search for an explanation for the ^•^O_2_^−^ selectivity using SA Pt co‐catalysts in photocatalysis some electrochemical work on O_2_ reduction on Pt electrodes may be helpful. As mentioned in the introduction, for electrocatalytic O_2_ reduction on bulk Pt, generally a four‐electron reaction to H_2_O takes place. A key feature of Pt SAs is that they do not provide a continuous Pt‐Pt bond which strongly favors lower (1 or 2) electron pathways.^[^ ^30^ ^]^ In turn, the selectivity of superoxide versus peroxide is based on theoretical considerations^[^ ^31^ ^]^ decided by the exact SAs coordination (substrate, defects), which impacts the energy barriers for the protonation steps necessary to form H_2_O_2_. Therefore, we also can deduce for photocatalysis that the presence of the SA Pt catalyzes the two‐electron pathway whereas the observed selectivity of superoxide formation over peroxide in our photocatalytic system must likely be attributed to the “specific configuration” of the Pt SAs on the titania surface. This precise positioning, achieved through reactive deposition, follows a “self‐homing” mechanism—where Pt SAs naturally anchor at the most reactive sites on the substrate until saturation.^[^ ^18^, ^32^ ^]^ These self‐anchored Pt SAs serve as highly active co‐catalytic centers, significantly enhancing electron transfer processes.

## 3 Conclusion

This study reveals a striking contrast between platinum single atoms (Pt SAs) and platinum nanoparticles (Pt NPs) in enhancing photocatalytic superoxide (^•^O_2_^−^) formation on titania in O_2_‐containing solutions. Pt SAs on titania, by facilitating a more efficient one electron transfer path, significantly boost the production of superoxide, a key reactive oxygen species in photocatalytic processes. In contrast, Pt NPs show minimal catalytic impact, highlighting the unique behavior of single atoms in tailoring photocatalytic reaction pathways at the atomic scale. These findings underscore a novel use of Pt SAs in photocatalytic applications. Practical implications are particularly in the use of photocatalysis for environmental remediation, pollutant degradation, and selective oxidation in organic synthesis where a superoxide oxidant is required. In a more general context, the results represent another example, where atomic scale co‐catalysts on a certain substrate and configuration can provide “unusual” reaction pathways and catalytic activity, making them highly interesting –in our case – for applications where the control of reactive oxygen species is crucial.

## Conflict Of Interest

The authors declare no conflict of interest.

## Supporting information



Supporting Information

## Data Availability

The data that support the findings of this study are available from the corresponding author upon reasonable request.
