# Supplementary material for: Platinum Single Atoms Strongly Promote Superoxide Formation in Titania‐Based Photocatalysis – Platinum Nanoparticles Don't
Source: Small. 2025 Feb 16;21(11):2412097. doi: 10.1002/smll.202412097 (PMC11922030; doi:10.1002/smll.202412097)
Supplement: Supplementary file 1 — Supporting Information [file SMLL-21-2412097-s001.docx]

Supporting Information

**Platinum Single Atoms Strongly Promote Superoxide Formation in Titania-Based Photocatalysis – Platinum Nanoparticles Don't**

*Yue Wang^a#^, Siming Wu^a#^, Giorgio Zoppellaro^b,c^,* *Zdeněk Baďura^b,c^, Patrik Schmuki^a,b*^*

^a^ Department of Materials Science and Engineering, Chair for Surface Science and Corrosion (WW4-LKO), Friedrich-Alexander-Universität Erlangen-Nürnberg, Martensstraße 7, 91058 Erlangen, Germany

^b^ Czech Advanced Technology and Research Institute, CATRIN, Regional Centre of Advanced Technologies and Materials (RCPTM), Palacky University in Olomouc, Šlechtitelů 11, 78371 Olomouc, Czech Republic.

^c^ CEET, Nanotechnology Centre, VŠB–Technical University of Ostrava, 17. listopadu 2172/15, 70800 Ostrava-Poruba, Czech Republic.

# Equal contributions

* Corresponding author: schmuki@ww.uni-erlangen.de

**Experimental Section**

**Materials**

Methanol (99.9%, Carl Roth), ethanol (99.8%, Carl Roth), 2-propanol (99.9%, Carl Roth), hydrogen peroxide (H_2_O_2_, 30%, Carl Roth), Nitrotetrazolium Blue chloride (NBT, Sigma-Aldrich) and H_2_PtCl_6_·6H_2_O (40.17% metallic Pt weight concentration, Metakem) were used without any additional purification. TiO_2_ rutile nanopowders (≈ 30 nm particle size, 99.9%) was purchased from US Research Nanomaterials, Inc.

**Pt Deposition**

Pt single atoms (SAs) decorated TiO_2_ rutile powders were prepared by using an adopted “reactive deposition” approach with H_2_PtCl_6_·6H_2_O as the Pt source.^[1]^ The powders (0.5 g L^-1^) were dispersed in a quartz cell containing Ar purged H_2_PtCl_6_·6H_2_O aqueous solutions (0.005 mM, 0.02 mM or 2 mM), and then the suspension was kept in dark with continuous stirring for 1 h. Upon completion, the powders were collected and washed three times by centrifuging and rinsing with DI water, then dried at 70 ^o^C in air.

For comparison, TiO_2_ rutile powders were also decorated with metallic Pt nanoparticles (NPs) using a conventional photodeposition process.^[2]^ In this approach, the rutile powders (10 mg or 20 mg) were suspended in 10 mL of methanol:water (1:1 volume ratio) mixture containing 0.03 mM or 0.05 mM H_2_PtCl_6_ for the controlled loading of Pt. The suspensions were then purged with Ar and illuminated by a 365 nm LED (600 mW cm^-2^) for 3 h. After illumination, the nanoparticles were washed via centrifuging and rinsing with deionized water three times, followed by drying in air at 70 °C.

**Characterization**

High-angle annular dark-field scanning transmission electron microscopy (HAADF-STEM) images and EDX mapping of the samples were acquired by a probe-corrected scanning transmission electron microscope (Thermo Fisher Scientific Spectra 200). The morphology of the samples was investigated by field-emission scanning electron microscope (FE-SEM, S-4800, Hitachi). The crystalline structure of the samples was determined by X-ray diffraction (XRD, X’pert Philips MPD with a Panalytical X’celerator detector) by means of graphite monochromatized Cu Kα radiation (wavelength 1.5406 Å). The chemical composition of samples was analyzed by X-ray photoelectron spectroscopy (XPS, PHI 5600). All XPS spectra were shifted to a standard Ti2p binding energy of 458.5 eV and the peak deconvolution was carried out by MultiPak software. Pt loading was further determined by electro thermal atomization-atomic absorption spectroscopy (ETA-AAS) using a graphite furnace with a ContrAA 600 Spectrometer (Analytik Jena AG) equipped with a high-resolution Echelle double monochromator and a continuum radiation source (Xe lamp).

**NBT assay**

NBT assays were carried out to assess the ^•^O_2_^−^ production in bare TiO_2_ and TiO_2_ decorated with Pt as SAs or NPs. The NBT maximum absorption peak is situated far from the wavelength of the monochromatic 365 nm LED light source, resulting in negligible absorption at this wavelength. Hence, employing NBT for superoxide detection does not conflict with light absorption issues. Notably, the reaction between NBT and ^•^O_2_^−^ resulted in the formation of a dark precipitate insoluble in aqueous solutions.^[3]^ The comparison of ^•^O_2_^−^ production of various samples was executed measuring the decreased intensity of the absorbance of NBT in the solution.

Absorbance spectra of the solutions in the wavelength range of 200−800 nm were measured using a UV/VIS/NIR spectrometer (Lambda 950, Perkin Elmer). The spectrometer was equipped with a tungsten-halogen lamp for UV light. The solutions were placed in transparent quartz cuvettes (Hellma Analytics), and the relative color intensity was correlated with the maximum absorbance peak at 260 nm.

The NBT assay, with a 4% 2-propanol solution containing 40 μM NBT as starting concentration, was performed in air. The suspended photocatalyst (bare TiO_2_, TiO_2_ decorated with Pt SAs or Pt NPs) was placed in a quartz reactor under vigorous stirring. To ensure the establishment of an adsorption/desorption equilibrium between the sample and NBT, the sample was immersed in the NBT solutions and kept in the dark for 1 hour before illumination (Figure S5), and then illuminated with an LED (λ = 365 nm, power density of 65 mW cm^-2^, exposure area = 0.785 cm^2^).

After different UV illumination times (10, 30, 60 min), the suspension was centrifuged, and 3 ml of supernatant was taken for UV-vis spectra measurement. After analysis, the extracted portion was returned to maintain the original reaction volume, and the total irradiation time was 60 minutes.

**EPR**

Continuous wave electron paramagnetic resonance spectra were recorded on a JEOL JES-X-320 spectrometer (JEOL, Tokyo, Japan) operating at the X-band frequency (≈ 9.08 GHz) equipped with a variable-temperature controller (He, N_2_) ES-CT470 apparatus. Highly pure quartz tubes were employed (Suprasil, Wilmad, ≤0.5 OD) as holders for glass capillary tubes (WHEATON^®^ 851321, 1−5 μL) loaded with water solutions containing 2,2,6,6, tetramethylpiperidine-*N*-oxyl radical (TEMPO) with and without the presence of the TiO_2_ catalysts. The TEMPO radical was purchased from Sigma-Aldrich (CAS N^o^: 2564-83-2, 98% purity).

For the *in-operando* measurements under UV-light irradiation a HeCd laser operating @ 325 nm was used (200 mW source, 40 mW/cm^2^ on the sample when loaded inside the cavity resonator) by direct fitting through optical wire the light source into the dedicated optical window of the resonator. Experimental temperature was set to *T* = 293 K in all measurements, modulation frequency to 100 kHz, time constant to 30 ms, 0.3 mT modulation width and 1 min acquisition time. Microwave power was selected to low values (0.3−0.9 mW) to avoid saturation effects during signal acquisition. In all EPR measurements the concentration of TEMPO radical dissolved in neat water (Sigma-Aldrich, HPLC grade, CAS Number: 7732-18-5) was kept at a constant concentration (1.95 × 10^-4^ M) and the concentration of the various TiO_2_ catalysts dispersed in water containing O_2_ was kept within the range 0.25−0.30 mg/mL. Analysis of the EPR spectra were obtained by performing double integration of the EPR signal intensities divided by the square root of the applied microwave power (*P*).

Within the series of experiments, the samples (only TEMPO in water, TEMPO plus catalysts in water) were at first recorded under dark conditions (*t* = 0) and then the radical oxidation was observed under light irradiation by monitoring *in situ* the variation of the TEMPO EPR signal intensities *vs* irradiation time. The double integrated EPR signal intensities divided by the root of power at *t* = 0 ([C] = 1.95 × 10^-4^ M) provided the normalization conditions from which all the other values recorded at various times (@325 nm) were compared. The equation used to simulate the observed trends in signal decrease of the TEMPO radical upon scavenging ROS species was the second-order kinetic, with numerical treatments that followed Equations (1) – (4).

Eq. (1) ∫∫EPR signal intensity / √*P* = [TEMPO],

where [TEMPO] at *t* = 0 (defined as [TEMPO]_0_) corresponds to the double integrated EPR signal area recorded under dark conditions

Eq. (2) – d[TEMPO] = *k* [TEMPO]^2^

Eq. (3) 1/[TEMPO] = 1/[TEMPO]_0_ + *kt*

The double integrated signal intensities under light irradiation were then compared to the value obtained under dark conditions such as:

[∫∫EPR signal intensity / √*P*]_t = x_ / [∫∫EPR signal intensity / √*P*]_t = 0_

Therefore, Eq. 3, after such normalization condition, can be expressed in the simplified form (4):

Eq. (4) 1/[TEMPO] = 1 + *kt*

**Figures:**


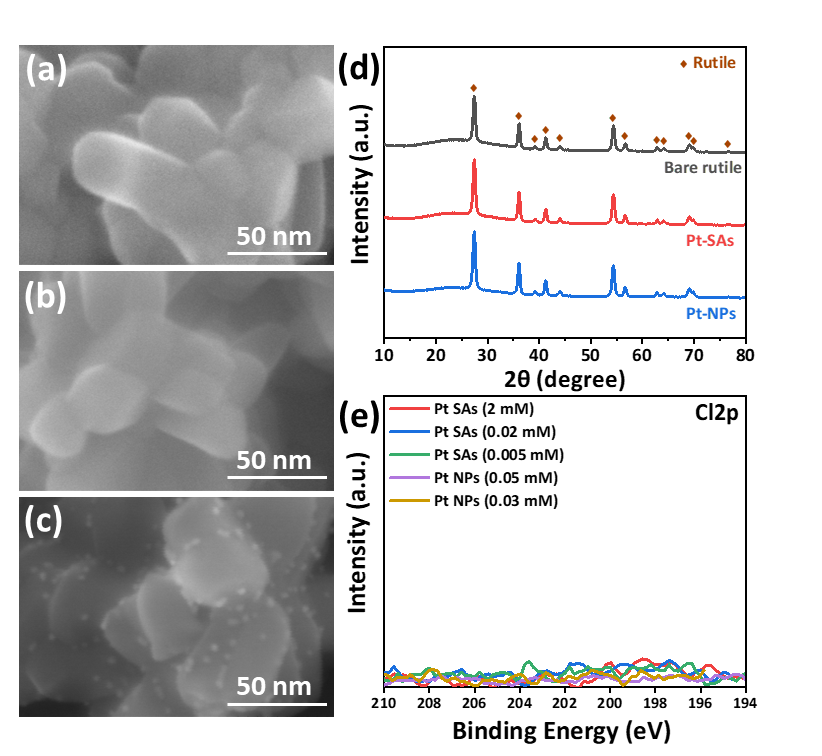


**Figure S1.** SEM images of rutile powders decorated with Pt SAs by reactive deposition from **(a)** 2 mM H_2_PtCl_6_ and **(b)** 0.005 mM H_2_PtCl_6_, and **(c)** decorated with Pt NPs by photodeposition from 0.03 mM H_2_PtCl_6_, respectively. **(d)** XRD patterns of bare rutile powders, after decorated with Pt SAs by reactive deposition and decorated with Pt NPs by photodeposition, respectively. **(e)** XPS Cl2p spectra of rutile powders after reactive deposition and photodeposition from different concentrations of H_2_PtCl_6_ solution.


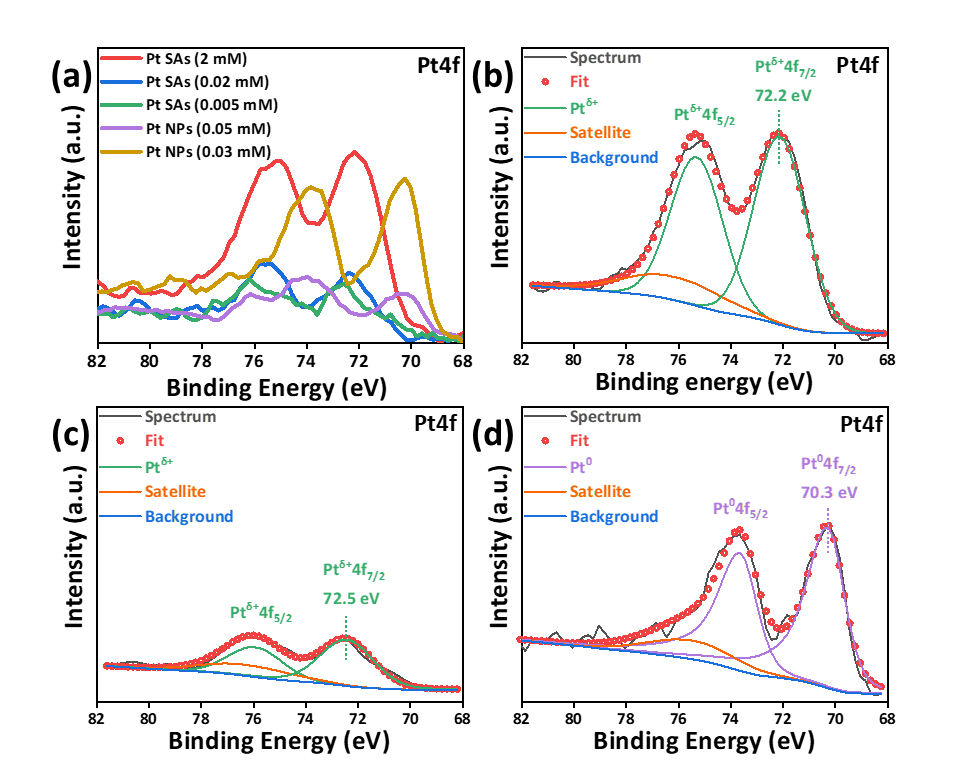


**Figure S2.** **(a)** XPS Pt4f spectra of rutile powders after reactive deposition and photodeposition from different concentrations of H_2_PtCl_6_ solution. Corresponding fitting details of rutile powders decorated with Pt SAs by reactive deposition from **(b)** 2 mM H_2_PtCl_6_ and **(c)** 0.005 mM H_2_PtCl_6_, and **(d)** decorated with Pt NPs by photodeposition from 0.03 mM H_2_PtCl_6_, respectively.


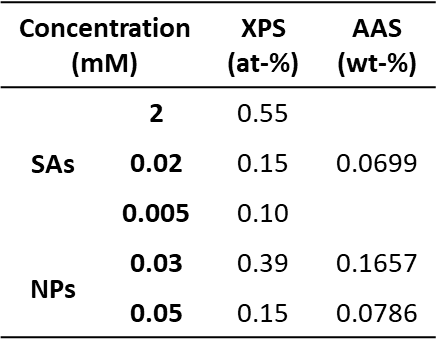


**Table S1.** Table of Pt loading on rutile powders prepared by reactive deposition and photodeposition from different concentrations of H_2_PtCl_6_ solution.


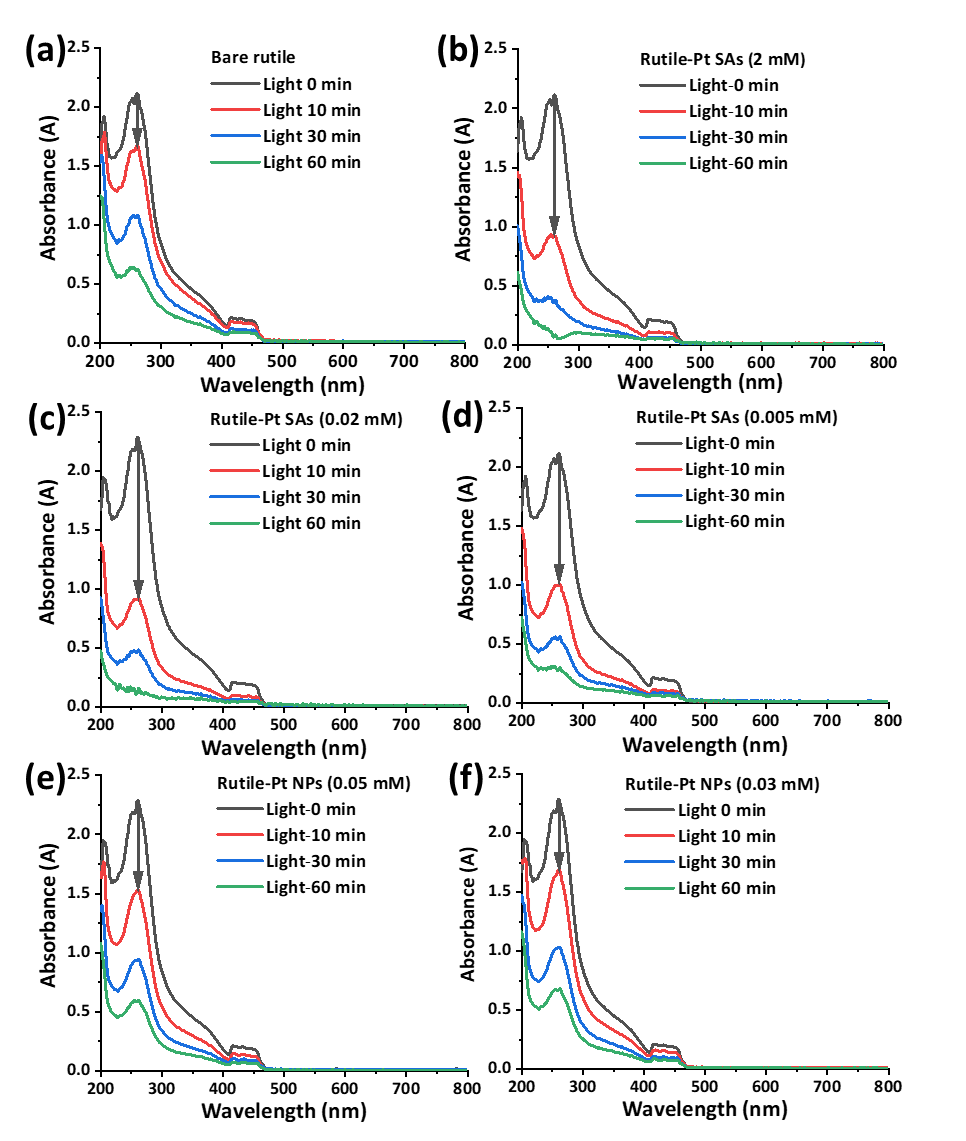


**Figure S3.** UV-vis absorption spectra of NBT assay executed with **(a)** bare rutile powders, **(b-d)** rutile powders decorated with Pt SAs by reactive deposition and **(e, f)** rutile powders decorated with Pt NPs by photodeposition, respectively.


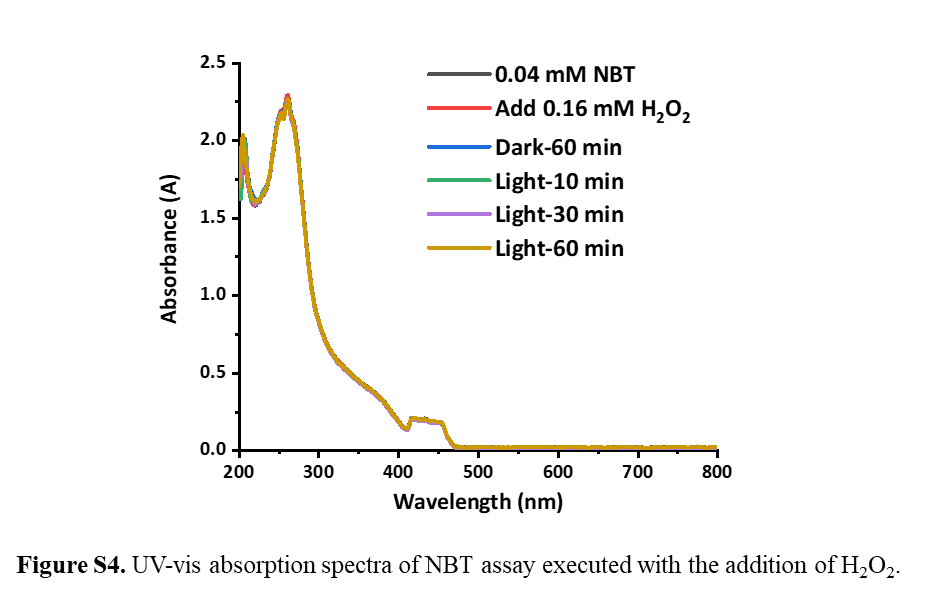


**Figure S4.** UV-vis absorption spectra of NBT assay executed with the addition of H_2_O_2_.


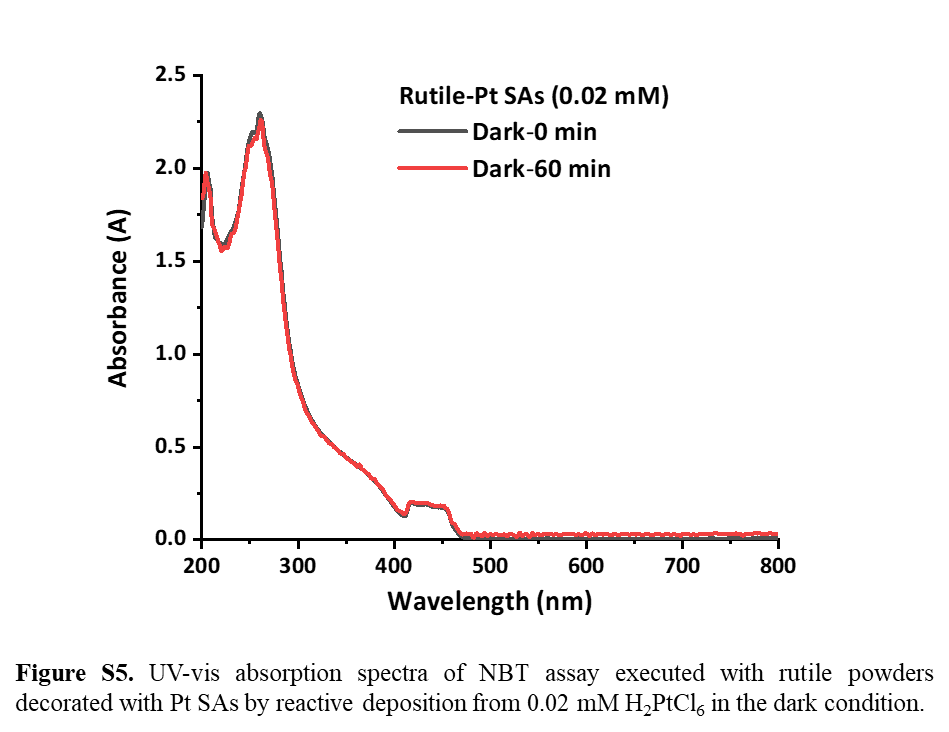


**Figure S5.** UV-vis absorption spectra of NBT assay executed with rutile powders decorated with Pt SAs by reactive deposition from 0.02 mM H_2_PtCl_6_ in the dark condition.

**References**

[1] a) Y. Wang, S. Qin, N. Denisov, H. Kim, Z. Bad’ura, B. B. Sarma, P. Schmuki, *Adv. Mater.* **2023**, *35*, 2211814;

b) Y. Wang, N. Denisov, S. Qin, D. S. Gonçalves, H. Kim, B. B. Sarma, P. Schmuki, *Adv. Mater.* **2024**, *36*, 2400626;

c) X. Zhou, Y. Wang, N. Denisov, H. Kim, J. Kim, J. Will, E. Spiecker, A. Vaskevich, P. Schmuki, *Small* **2024**, 2404064;

d) S.-M. Wu, L. Wu, N. Denisov, Z. Badura, G. Zoppellaro, X.-Y. Yang, P. Schmuki, *J. Am. Chem. Soc.* **2024**, *146*, 16363;

e) S. Qin, J. Will, H. Kim, N. Denisov, S. Carl, E. Spiecker, P. Schmuki, *ACS Energy Lett.* **2023**, *8*, 1209;

f) N. Denisov, S. Qin, J. Will, B. N. Vasiljevic, N. V. Skor odumova, I. A. Pašti, B. B. Sarma, B. Osuagwu, T. Yokosawa, J. Voss, J. Wirth, E. Spiecker, P. Schmuki, *Adv. Mater.* **2023**, *35*, 2206569.

[2] a) S. Qin, N. Denisov, B. B. Sarma, I. Hwang, D. E. Doronkin, O. Tomanec, S. Kment, P. Schmuki, *Adv. Mater. Interfaces* **2022**, *9*, 2200808;

b) S. Qin, N. Denisov, J. Will, J. Kolařík, E. Spiecker, P. Schmuki, *Solar RRL* **2022**, *6*, 2101026.

[3] H. Goto, Y. Hanada, T. Ohno, M. Matsumura, *J. Catal.* **2004**, *225*, 223.
